# Supplementary material for: Genome-wide DNA methylation is predictive of outcome in juvenile myelomonocytic leukemia
Source: Nat Commun. 2017 Dec 19;8:2127. doi: 10.1038/s41467-017-02178-9 (PMC5736624; doi:10.1038/s41467-017-02178-9)
Supplement: Supplementary file 1 — Supplementary Information [file 41467_2017_2178_MOESM1_ESM.pdf]

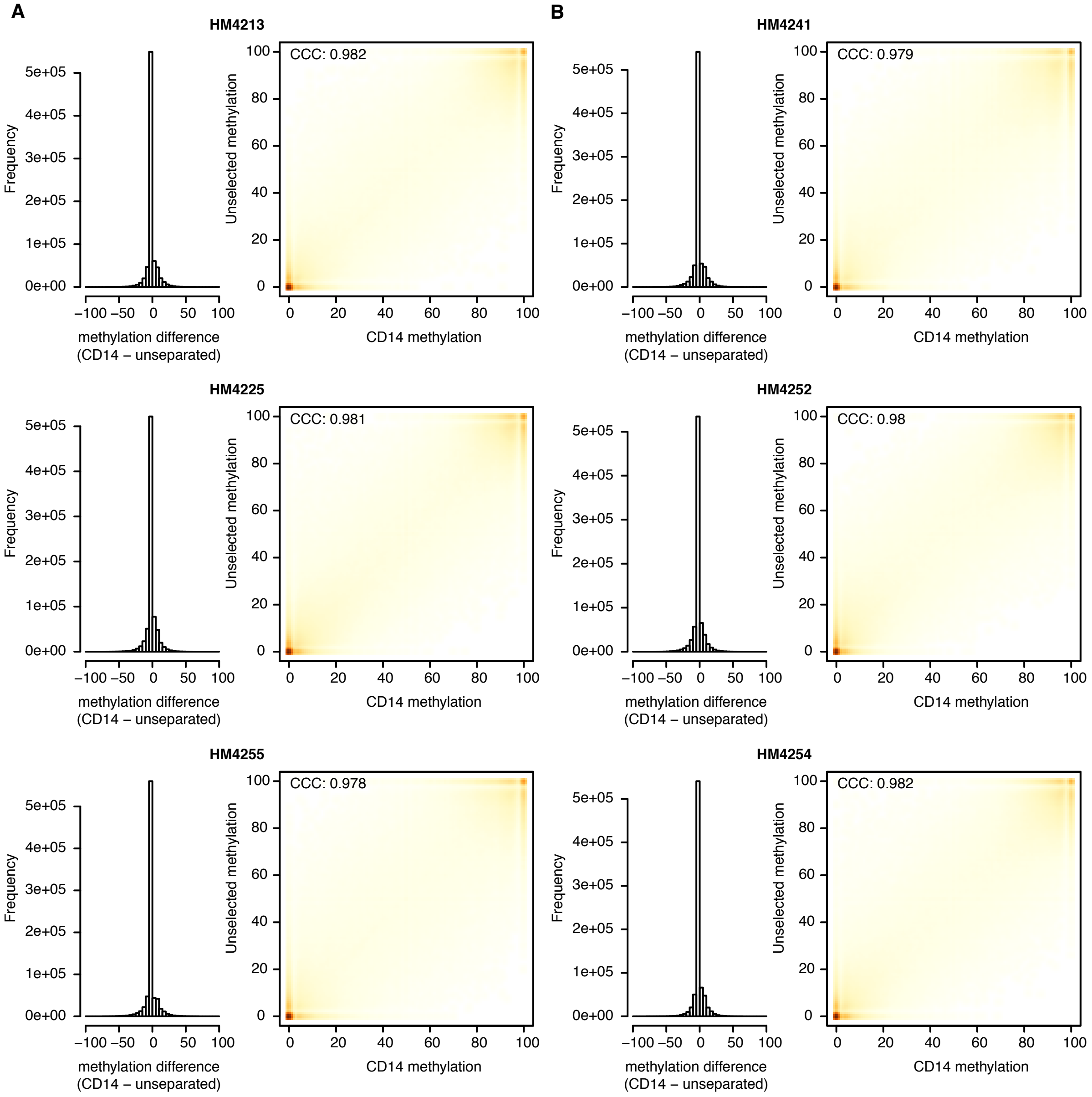

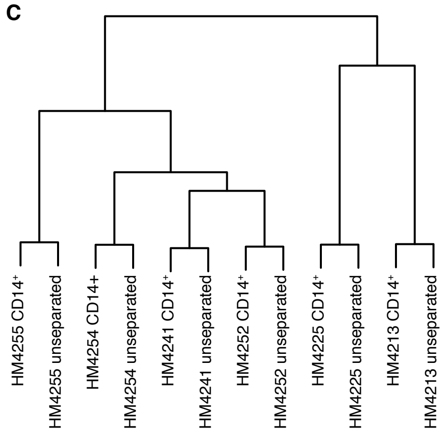

Supplementary Figure 1. Pilot methylation study results. (A) Concordance plots for three JMML patients comparing $CD14^+$ and unselected cells from the same patients. (B) Concordance plots for three age-appropriate controls comparing $CD14^+$ and unselected cells from the same subjects. (C) unsupervised clustering analysis of all six subjects using $CD14^+$ and unselected cells.

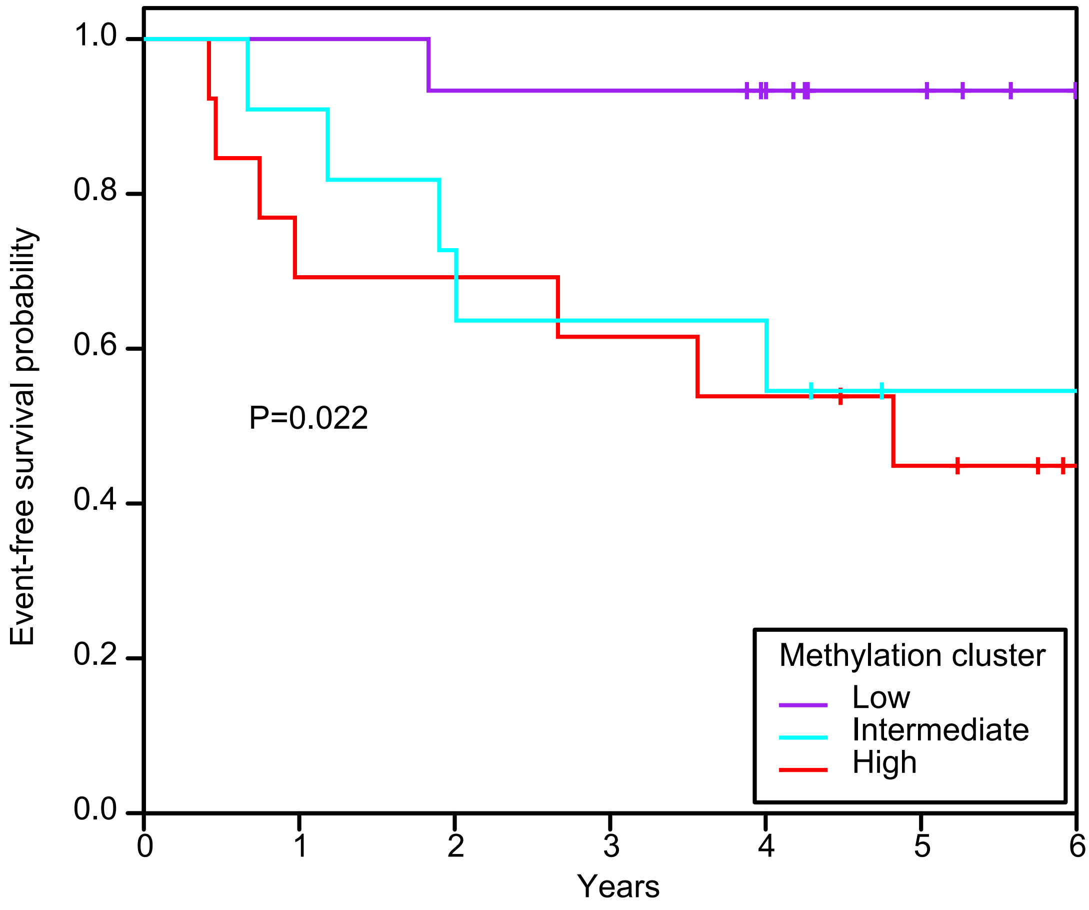

Supplementary Figure 2. Event Free Survival (EFS) based on methylation cluster. Patients with different methylation cluster designations had distinct outcomes (p=0.0039).

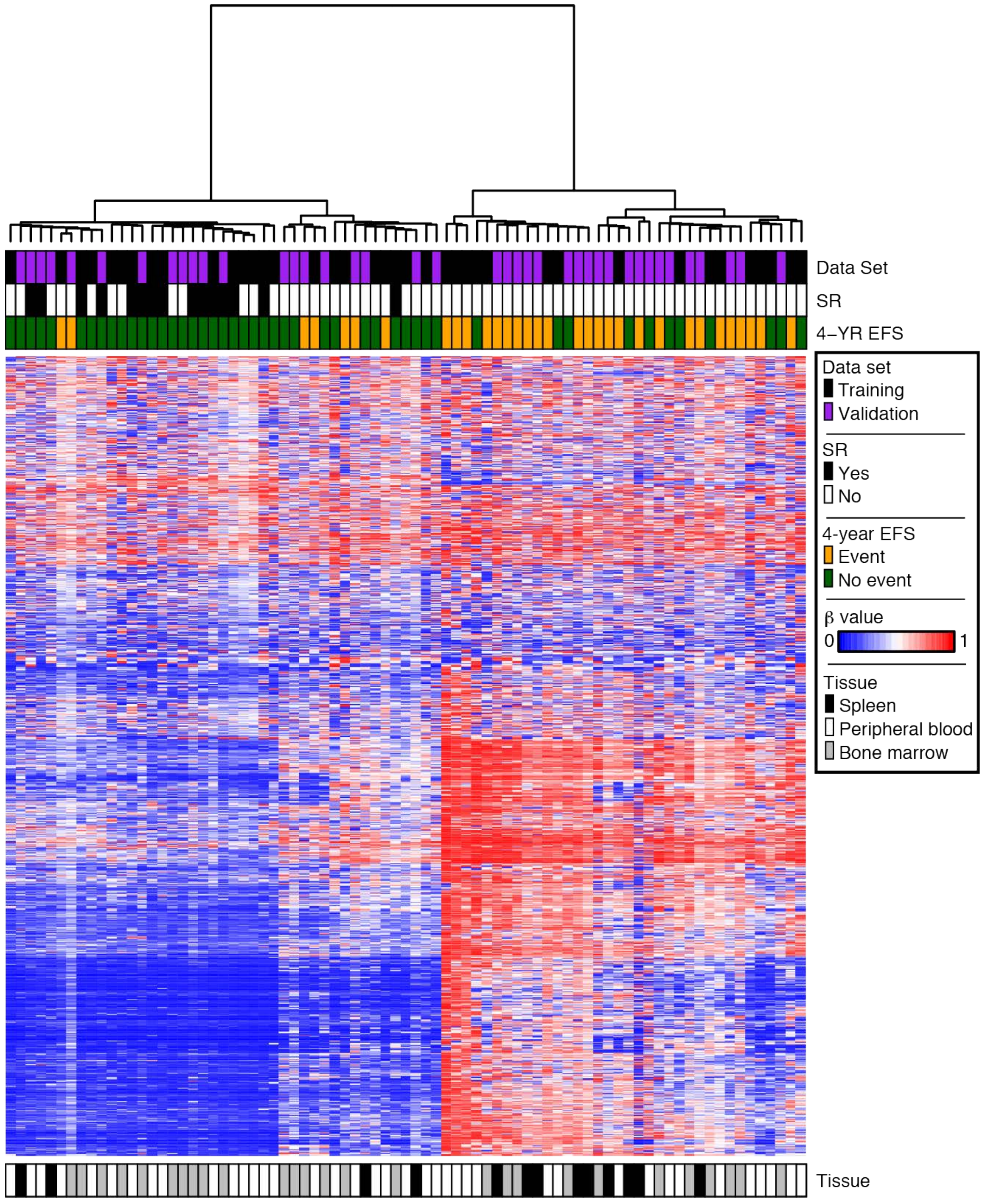

Supplementary Figure 3. **Unsupervised clustering analysis of all JMML patients.** Seventy-nine patients from both the discovery and validation cohorts are included. Tissue source for each sample is displayed on the bottom of the figure.

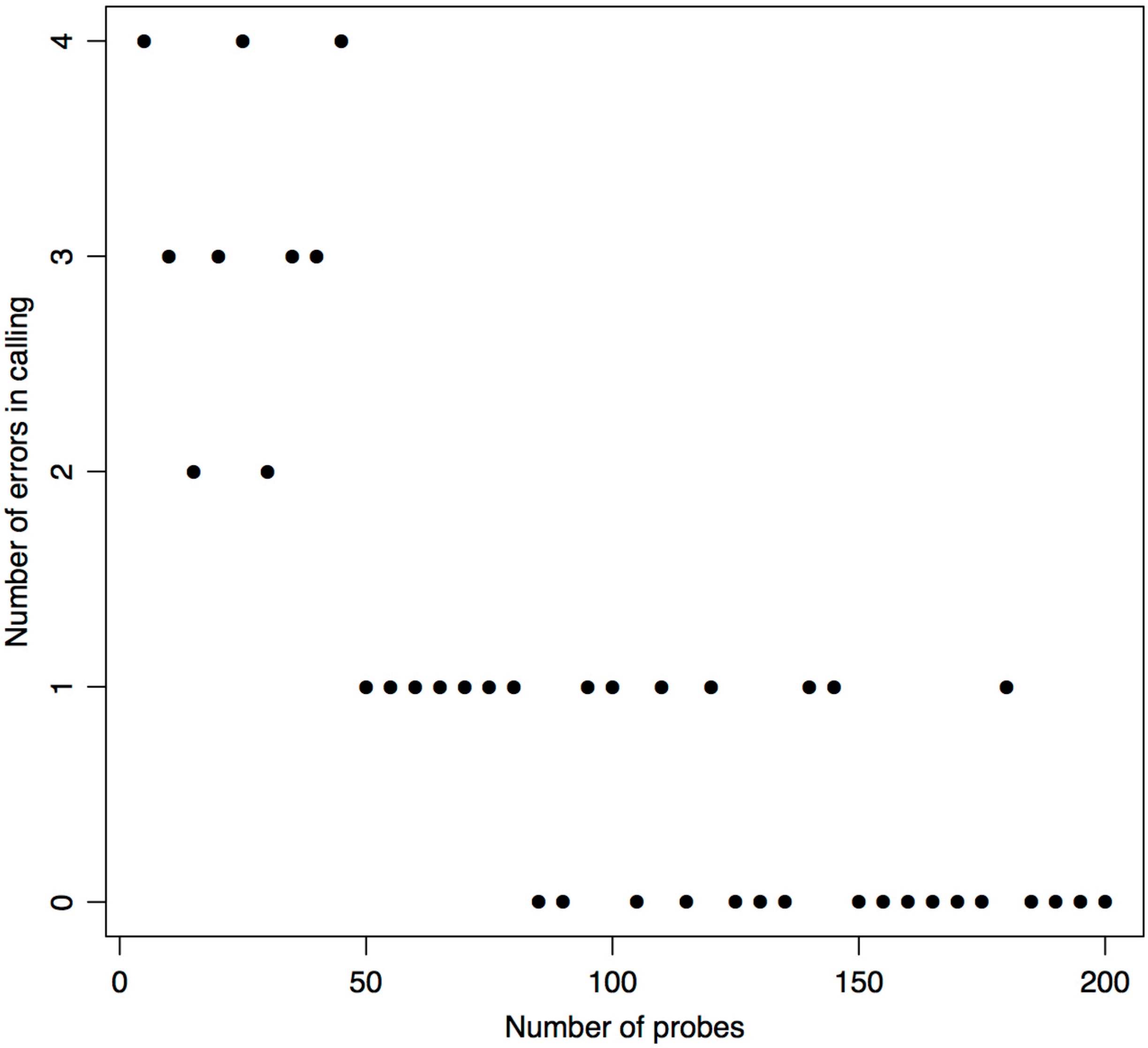

Supplementary Figure 4. Minimum number of CpGs required to distinguish low/intermediate/high methylation clusters. Using at least 50 CpG probes, the number of errors in classifying the validation cohort went down to either 0 or 1.

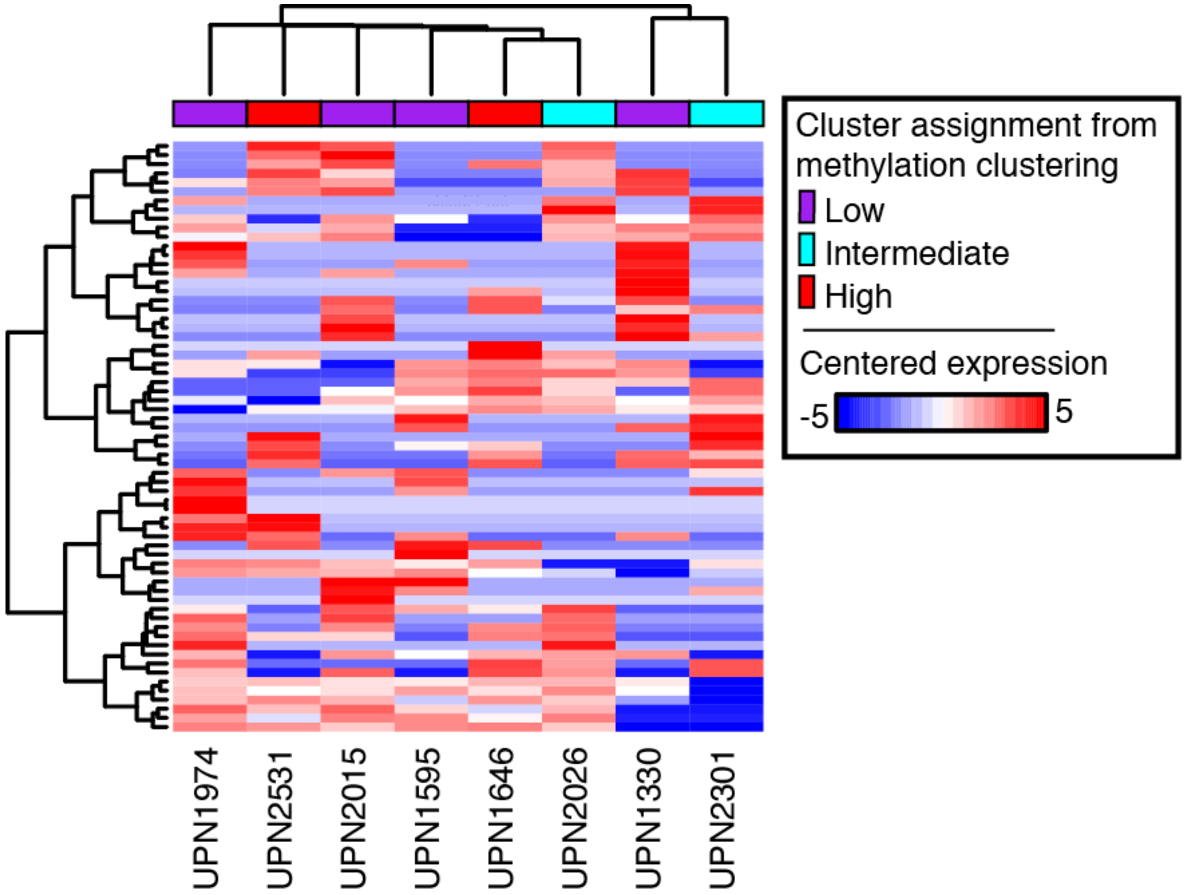

Supplementary Figure 5. **Unsupervised clustering using RNA sequencing data.** We clustered the expression data based on the genes that annotated to the most variable CpG probes, JMML patients did not cluster into the low, intermediate and high clusters seen with DNA methylation.

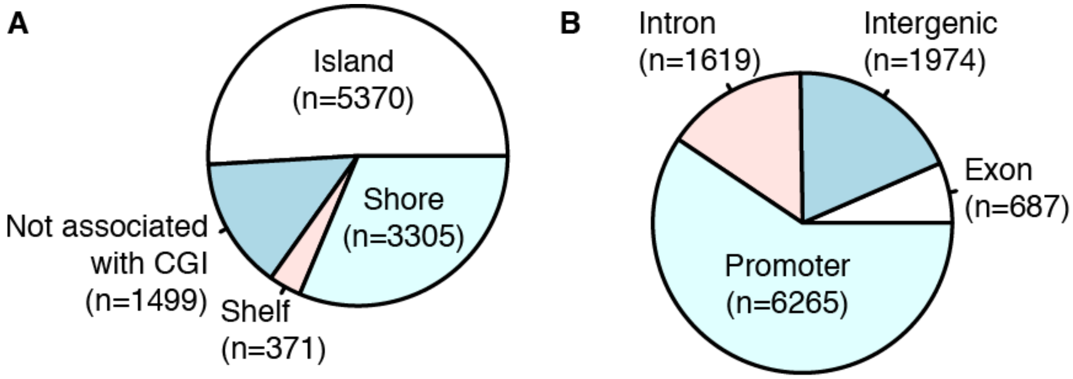

Supplementary Figure 6. **Distribution of significantly differentially methylated CpG sites.**The distribution of the 10,545 CpG sites that were differentially methylated between patients that do or do not have an event relative to (A) CpG islands and (B) gene space. When a CpG site was associated with multiple genes/transcripts, priority was assigned as follows: promoter > exon > intron > intergenic.

Supplementary Table 1. Mutation status in progenitor cells.

UPN	Tissue Source	Spontaneous Remission	Protein Alteration	HSC	MPP	CMP	GMP
1330	BM	Yes	NRAS p.G12S	Yes	Yes	Yes	Yes
1383	BM	No	SETBP1 p.G870S	Yes	Yes	Yes	Yes
1778	BM	Yes	CBL p.Y371N	Yes	Yes	Yes	Yes
2531	BM	No	PTPN11 p.E69K	Yes	Yes	Yes	Yes

Supplementary Table 2. Allelic fraction of driver mutations in different cellular subsets.

Patient	Mutation	Allelic Fraction in Mononuclear Cells	Allelic Fraction in CD3+ Cells	Allelic Fraction in CD14+ Cells	Allelic Fraction in CD19+ Cells	Allelic Fraction in CD34+ Cells
UPN2570	PTPN11 p.D61Y	0.44	0.17	0.50	0.48	0.44
UPN2567	KRAS p.G12D	0.40	0.06	0.49	0.26	0.45
UPN2566	KRAS p.G12D	0.45	0.06	0.52	0.47	0.44

Supplementary Table 3. Clinical, genomic and outcome characteristics of discovery cohort.

No. Patients	39
Median Age, Months (range)	17 (1-87)
Gender (%)	
Male	28 (72)
Female	11 (28)
Blood counts	
Median WBC Count x10^9/L (range)	28.3 (9-109)
Median Platelet Count x10^9/L (range)	59.0 (10-189)
Fetal hemoglobin (%)	
Elevated	21 (54)
Not Elevated	17 (44)
Unknown	1 (3)
GM-CSF Hypersensitivity (%)	
Yes	25 (64)
No	7 (18)
Not Done	7 (18)
Monosomy 7 (%)	
Yes	2 (5)
No	37 (95)
Driver Mutation Status (%)	
PTPN11	13 (33)
NF1	8 (21)
NRAS	7 (18)
KRAS	6 (15)
CBL	6 (15)
SH2B3	1 (3)
Secondary Mutation Status (%)	
SETBP1	5 (13)
ASXL1	2 (5)
JAK3	2 (5)
DNMT3A	1 (3)
GATA2	1 (3)
PTPN11	1 (3)
ZRSR2	1 (3)
SH2B3	1 (3)
Treatment (%)	
Transplant	31 (80)
Observation	7 (18)
Chemotherapy	1 (3)
Event Free Status (%)	
Remission	25 (64)
Relapse	14 (36)
Overall Outcome (%)	
Alive	27 (70)
Deceased	12 (31)

Supplementary Table 4. Secondary mutations and cluster status.

UPN	Driver Mutation(s)	Driver Allelic Burden	Secondary Mutation(s)	Secondary Mutation Allelic Burden(s)	Methylation Cluster
UPN0906	PTPN11 p.E76K	0.57	ASXL1 p.E727*	0.35	High
UPN0969	NF1 p.T1972fs	0.88	NRAS p.G12V	0.40	High
UPN1383	NF1 p.R681*	0.59	SETBP1p.G870S	0.12	Intermediate
UPN1431	KRAS p.Q61L	0.49	SETBP1 p.D868N	0.48	High
UPN1447	PTPN11 p.E76k	0.30	DNMT3A p.G707fs	0.16	High
UPN1484	PTPN11p.G60R	0.49	NF1 p.T676fs, NF1 p.R1534*	0.5, 0.06	High
UPN1603	PTPN11 p.G503V	0.51	JAK3 p.R657Q	0.51	High
UPN1646	NRAS p.G12D	0.44	ZRSR2 p.G179E	0.90	High
UPN1711	NF1 p.N339fs	0.99	SETBP1 p.G870S, ASXL1 pH630fs, GATA2 p.N317S	0.56, 0.45, 0.58	High
UPN1875	PTPN11 p.E76K	0.35	SETBP1 p.I871T	0.34	High
UPN2301	KRAS p.G13D	0.19	SETBP1 p.D868N	0.29	Intermediate
UPN2531	PTPN11 p.E69K	0.39	SH2B3 p.W262*, SH2B3 p.H414fs	0.35, 0.40	High

Supplementary Table 5. Multivariable analysis.

**Multiivariable Analysis**	**EFS from date of diagnosis**			
	N	OR	95% CI	p
Age at diagnosis (months)				0.12
≤12 months	17	1		
>12 months	22	3.594	0.36-44.974	
Cluster				**0.032**
Low	15	1		
Intermediate	11	2.312	0.069-79.599	
High	13	7.94	0.833-186.27	
Somatic Mutations at Diagnosis				**0.018**
<=1	27	1		
>1	12	7.121	0.725-169.968	

Supplementary Table 6. Characterstics of validation cohort.

UPN	Array ID	Treatment	Outcome	Mutation	Tissue	Cluster
1578	200397860087_R02C02	HSCT	Remission	NF1	PB	Intermediate
1786	200277210006_R01C01	HSCT	Remission	None	PB	High
4233	9444374126_R05C01	HSCT	Relapse	NF1	Spleen	High
4421	9444374124_R02C02	HSCT	Remission	None	Spleen	Low
4587	9444374124_R03C01	HSCT	Relapse	NF1	Spleen	High
4645	9444374124_R04C01	HSCT	Relapse	NRAS	Spleen	Intermediate
5377	9444374126_R06C01	HSCT	Relapse	PTPN11	Spleen	High
5417	9444374126_R01C02	HSCT	Relapse	PTPN11	Spleen	High
5628	9444374124_R03C02	HSCT	Remission	KRAS	Spleen	Low
6162	9444374124_R05C01	HSCT	Relapse	KRAS	Spleen	High
6265	9444374126_R03C01	HSCT	Remission	None	Spleen	High
6307	9444374124_R01C01	HSCT	Remission	PTPN11	Spleen	Intermediate
6624	200397860087_R04C01	None	Remission	CBL	BM	Low
12600	9444374126_R02C02	HSCT	Relapse	KRAS	Spleen	High
99889	200078650239_R01C02	HSCT	Remission	KRAS	BM	Intermediate
105214	200397860086_R01C01	None	Remission	NRAS	BM	Low
107530	200277210006_R02C01	None	Remission	CBL	BM	Low
112029	200277210006_R03C01	HSCT	Relapse	NRAS	BM	High
122865	200277210006_R01C02	None	Remission	CBL	BM	Low
125513	200397860089_R02C01	HSCT	Relapse	PTPN11	BM	High
140211	200078650239_R03C02	HSCT	Remission	NF1	BM	Intermediate
140251	200397860100_R01C01	HSCT	Relapse	KRAS	BM	Low
141302	200397860086_R05C01	HSCT	Relapse	PTPN11	BM	Intermediate
143828	200078650182_R06C02	None	Remission	NRAS	BM	Low
153038	200277210018_R03C01	HSCT	Remission	KRAS	BM	Intermediate
153843	200277210018_R04C01	HSCT	Remission	None	BM	Intermediate
161112	200078650182_R05C02	HSCT	Remission	PTPN11	PB	High
163365	200277210018_R01C02	HSCT	Relapse	PTPN11	BM	Intermediate
163735	9444374126_R01C01	HSCT	Remission	KRAS	Spleen	Intermediate
163946	200277210018_R02C02	HSCT	Relapse	PTPN11	BM	High
167550	9444374124_R06C02	HSCT	Relapse	KRAS	Spleen	High
175209	200078650239_R03C01	HSCT	Remission	PTPN11	BM	High
177314	200397860089_R04C02	None	Remission	NRAS	PB	Low
178394	200277210055_R02C01	HSCT	Relapse	None	BM	High
185028	200397860089_R03C01	HSCT	Relapse	PTPN11	BM	High
186428	200277210006_R04C01	HSCT	Remission	NRAS	BM	Low
189363	200078650239_R01C01	HSCT	Relapse	NRAS	PB	Intermediate
191048	200397860089_R04C01	HSCT	Relapse	PTPN11	BM	High
195753	200397860089_R02C02	None	Remission	NRAS	PB	Low
206021	200277210044_R06C02	HSCT	Remission	NRAS	BM	Low

Supplementary Table 7. KEGG Pathway Enrichment Using Only DNA Methylation Data.

ko01100 Metabolic pathways (70)

ko04080 Neuroactive ligand-receptor interaction (60)

ko05200 Pathways in cancer (47)

ko04024 cAMP signaling pathway (34)

ko04020 Calcium signaling pathway (32)

ko05202 Transcriptional misregulation in cancer (28)

ko04360 Axon guidance (27)

ko04151 PI3K-Akt signaling pathway (25)

ko04010 MAPK signaling pathway (24)

ko04014 Ras signaling pathway (23)

ko04022 cGMP-PKG signaling pathway (23)

Supplementary Table 8. KEGG Pathway Enrichment using Overlapping LIMMA Analyses.

ko05202 Transcriptional misregulation in cancer (3)

ko04014 Ras signaling pathway (2)

ko05200 Pathways in cancer (2)

ko04914 Progesterone-mediated oocyte maturation (1)

ko04550 Signaling pathways regulating pluripotency of stem cells (1)

ko05165 Human papillomavirus infection (1)

ko04514 Cell adhesion molecules (CAMs) (1)

ko04024 cAMP signaling pathway (1)

ko05212 Pancreatic cancer (1)

Supplementary Table 9. Individual clinical characteristics of discovery cohort.

UPN	Array ID	Treatment	Outcome	Mutation	Tissue	Cluster
UPN0424	9761749076_R01C01	HSCT	Remission	*PTPN11*	BM	Low
UPN0647	9761749080_R02C01	HSCT	Remission	*NRAS*	BM	High
UPN0868	9482801069_R01C01	HSCT	Remission	*PTPN11*	PB	Intermediate
UPN0906	9482801069_R02C01	HSCT	Relapse	*PTPN11*	PB	High
UPN0969	9761749076_R02C01	HSCT	Relapse	*NF1*	PB	High
UPN1043	9482801069_R03C01	HSCT	Relapse	*PTPN11*	PB	Low
UPN1142	9482801069_R04C01	HSCT	Remission	*PTPN11*	PB	Intermediate
UPN1199	9482801069_R05C01	HSCT	Remission	*KRAS*	PB	Intermediate
UPN1312	9482801069_R06C01	HSCT	Relapse	*KRAS*	PB	Intermediate
UPN1330	9482801069_R01C02	None	Remission	*NRAS*	PB	Low
UPN1333	9761749076_R05C01	HSCT	Relapse	*CBL*	PB	Intermediate
UPN1348	9761749080_R03C01	None	Remission	*KRAS*	BM	Intermediate
UPN1383	9761749087_R01C01	HSCT	Relapse	*NF1*	BM	Intermediate
UPN1431	9482801069_R02C02	HSCT	Relapse	*KRAS*	PB	High
UPN1447	9482801069_R03C02	HSCT	Relapse	*PTPN11*	PB	High
UPN1484	9761749080_R04C01	HSCT	Relapse	*PTPN11*	PB	High
UPN1550	9482801069_R04C02	None	Remission	*NRAS*	PB	Low
UPN1595	9482801069_R05C02	HSCT	Remission	*NRAS*	PB	Low
UPN1603	9761749080_R05C01	HSCT	Remission	*PTPN11*	PB	High
UPN1646	9761749087_R02C01	HSCT	Remission	*NRAS*	PB	High
UPN1711	9482801024_R01C01	HSCT	Relapse	*NF1*	PB	High
UPN1740	9482801024_R02C01	HSCT	Remission	*KRAS*	PB	High
UPN1778	9482801024_R03C01	None	Remission	*CBL*	BM	Low
UPN1804	9761749080_R06C01	None	Remission	*NF1*	PB	Low
UPN1875	9761749087_R03C01	HSCT	Relapse	*PTPN11*	PB	High
UPN1941	9482801024_R04C01	HSCT	Remission	*PTPN11*	PB	High
UPN1970	9761749087_R04C01	HSCT	Remission	*SH2B3*	PB	Low
UPN1974	9761749087_R05C01	HSCT	Remission	*None*	PB	Low
UPN2015	9482801024_R05C01	None	Remission	*NRAS*	PB	Low
UPN2025	9482801024_R06C01	None	Remission	*CBL*	PB	Low
UPN2026	9761749087_R06C01	HSCT	Relapse	*PTPN11*	PB	Intermediate
UPN2056	9761749087_R01C02	None	Remission	*CBL*	PB	Low
UPN2099	9482801069_R06C02	HSCT	Remission	*NRAS*	PB	Low
UPN2178	9482801024_R01C02	HSCT	Remission	*CBL*	PB	Low
UPN2301	9761749087_R02C02	HSCT	Remission	*KRAS*	PB	Intermediate
UPN2309	9482801024_R03C02	HSCT	Remission	*CBL*	PB	Low
UPN2497	9482801024_R04C02	HSCT	Remission	*PTPN11*	PB	Intermediate
UPN2528	9482801024_R02C02	HSCT	Relapse	*NF1*	PB	Intermediate
UPN2531	9482801024_R05C02	HSCT	Relapse	*PTPN11*	BM	High

Abbreviations: UPN, universal patient number; HSCT, hematopoietic stem cell transplantation; BM, bone marrow; PB, peripheral blood.

Supplementary Table 10. Characteristics of samples used for pilot sorted cell methylation data shown in Supplementary Figure 1.

Patient	Disease Status	Sample	Tissue Source	Age (months)
UPN2570	JMML	HM4225	Peripheral Blood	26
UPN2567	JMML	HM4213	Peripheral Blood	6
UPN2566	JMML Healthy	HM4255	Peripheral Blood	23
UPN2576	Control Healthy	HM4241	Bone Marrow	12
UPN2205	Control Healthy	HM4252	Peripheral Blood	42
UPN2516	Control	HM4254	Bone Marrow	30

Supplementary Table 11. Characteristics of samples used for RNA sequencing shown in Supplementary Figure 6.

Patient	Disease Status	Sample	Methylation Cluster	Outcome
UPN1330	JMML	HM1989	Low	Remission
UPN1595	JMML	HM2414	Low	Remission
UPN1646	JMML	HM2494	High	Remission
UPN1974	JMML	HM3068	Low	Remission
UPN2015	JMML	HM3134	Low	Remission
UPN2026	JMML	HM3158	Intermediate	Relapse
UPN2301	JMML	HM3679	Intermediate	Remission
UPN2531	JMML	HM4155	High	Relapse
